# The Health and Well-Being of Older Adults with Dual Sensory Impairment (DSI) in Four Countries

**DOI:** 10.1371/journal.pone.0155073

**Published:** 2016-05-05

**Authors:** Dawn M. Guthrie, Anja Declercq, Harriet Finne-Soveri, Brant E. Fries, John P. Hirdes

**Affiliations:** 1 Department of Kinesiology & Physical Education and Department of Health Sciences, Wilfrid Laurier University, Waterloo, Ontario, Canada; 2 Katholieke Universiteit Leuven (Dutch), Brussels, Belgium; 3 National Institute for Health and Welfare, Ageing and Services Unit, Helsinki, Finland; 4 Institute of Gerontology, University of Michigan, Ann Arbor, Michigan, United States of America; 5 Health Systems Research and Geriatric Research, Education, and Clinical Center, Veterans Affairs Ann Arbor Healthcare System, Ann Arbor, Michigan, United States of America; 6 School of Public Health and Health Systems, University of Waterloo, Waterloo, Ontario, Canada; Banner Alzheimer's Institute, UNITED STATES

## Abstract

**Objectives:**

Dual sensory impairment (DSI) is a combination of vision and hearing impairments that represents a unique disability affecting all aspects of a person’s life. The rates of DSI are expected to increase due to population aging, yet little is known about DSI among older adults (65+). The prevalence of DSI and client characteristics were examined among two groups, namely, older adults receiving home care services or those residing in a long-term care (LTC) facility in four countries (Canada, US, Finland, Belgium).

**Methods:**

Existing data, using an interRAI assessment, were analyzed to compare older adults with DSI to all others across demographic characteristics, functional and psychosocial outcomes.

**Results:**

In home care, the prevalence of DSI across the four countries ranged from 13.4% to 24.6%; in LTC facilities, it ranged from 9.7% to 33.9%. Clients with DSI were more likely to be 85+, have moderate/severe cognitive impairment, impairments in activities of daily living, and have communication difficulties. Among residents of LTC facilities, individuals with DSI were more likely to be 85+ and more likely have a diagnosis of Alzheimer’s disease. Having DSI increased the likelihood of depression in both care settings, but after adjusting for other factors, it remained significant only in the home care sample.

**Conclusions:**

While the prevalence of DSI cross nationally is similar to that of other illnesses such as diabetes, depression, and Alzheimer’s disease, we have a limited understanding of its affects among older adults. Raising awareness of this unique disability is imperative to insure that individuals receive the necessary rehabilitation and supportive services to improve their level of independence and quality of life.

## Introduction

Dual sensory impairment (DSI), or deafblindness, is a combination of vision and hearing impairment that interferes with the person’s ability to acquire information and communicate with others.[1] DSI encompasses a spectrum of sensory loss and individuals may be completely deaf and completely blind or they may have some residual hearing and/or vision. The widely used Nordic definition states that “deafblindness is a combined vision and hearing disability. It limits activities of a person and restricts full participation in society to such a degree that society is required to facilitate specific services, environmental alterations and/or technology.”[2] The loss of functioning in one sense cannot be compensated for with the other sense, resulting in a distinct disability. Those with DSI, or deafblindness, can be broadly classified into two main groups based on when the sensory losses occurred: a) congenital (individuals who experience the onset of hearing and visual impairment before the age of two years, including onset at birth)[3]; and b) acquired (onset later in life). Common causes of congenital DSI include intra-uterine infections (e.g., congenital rubella syndrome),[4] congenital brain damage and chromosomal abnormalities such as CHARGE syndrome.[5, 6] Acquired DSI can be related to aging, post-natal infections/early childhood infections and acquired brain injury, [5, 7]however, Usher syndrome is the leading cause of DSI around the world.[8] Among older persons with DSI, vision loss is often a result of presbyopia, decreased pupil size, cataracts and glaucoma, [7, 9] and causes of hearing loss include age-related changes in the inner ear and a reduction in blood flow and loss of neurons that result in a diminished capacity within the central auditory system.[7]

In Canada, roughly 70,000 individuals (12+) have DSI.^1^ Prevalence estimates among older adults (65+) in North America range from 3% to 21%,[10, 11] while estimates across several European countries are typically between 6–7%,[12–15]and generally increase with age.[10] In the US, approximately 4.4 million older individuals experience some degree of DSI.[16] Population aging will result in a rising prevalence of age-related DSI.[17, 18]

On their own, both vision loss and hearing loss appear to contribute to negative outcomes in older adults such as higher rates of loneliness[6] or social isolation[12] and increased mortality related to heart disease.[13] There is little information on older adults with DSI since the literature focuses on individuals under 18. From the limited research to date, older persons with DSI appear at increased risk for reduced independence in activities of daily living (ADLs)[7, 11, 14, 19] and instrumental ADLs (IADLs),[14, 19, 20] cognitive impairment,[20–23] lower self-rated health,[21, 23, 24] all-cause mortality,[21] and social isolation.[25, 26]

A small number of studies among those aged 65+ have also shown an increased risk of depression among persons with DSI.[12, 14, 16, 20] However, these studies typically focused on a healthy, community-dwelling population and only two studies adjusted for multiple predictors of depression beyond DSI,[12, 16] limiting our understanding of the relationship between DSI and other risk factors.

Communication impairment is the main limitation associated with DSI. Although some individuals with DSI may have adjusted to a single sensory loss (e.g., with deafness they have become proficient in sign language), the onset of the second impairment has far-reaching implications for their preferred mode of communication (e.g., they may not have adequate vision to see a sign language interpreter). Communication difficulties can lead to frequent feelings of social ostracism,[24] fatigue (often thought of as the “third” disability), and embarrassment, especially during the initial onset of the condition.[27] Vision and hearing impairments make it difficult to navigate in unfamiliar surroundings and lead to a loss of independence.[28] DSI, especially for those with Usher syndrome, is also characterized by continued sensory losses over time, whereby the individual must continually adapt and change to meet these new challenges; these may result in changes in occupation or giving up working altogether.

With population aging, the rate of DSI will continue to increase; unaddressed, this will have important implications for the health and social services sectors. Older individuals with DSI may have other chronic health conditions and a need for health care services or residential care, but may also need specialized support and training related to the sensory impairment, which often fall under the umbrella of social services. Therefore, this disability requires multiple sectors of government and different types of providers to work together.

The main goals of this paper are: a) to understand the prevalence of DSI in two continuing care settings, home care and Long Term Care Facilities (LTCFs), across multiple countries in North America and Europe; b) to compare individuals with DSI to those without this impairment, to understand their complex needs; and c) to identify the relationship between DSI and depression after adjusting for other important covariates. We used existing data based on the interRAI family of instruments. InterRAI (www.interrai.org) is an international not-for-profit group of about 90 researchers, clinicians and health administrators from 35 countries. Its mandate is to develop and test standardized assessments to be used with frail and vulnerable populations. Instruments have been developed for a wide range of health and social service settings (e.g., LTCFs, inpatient and community mental health, community support services for intellectual disability, acute hospitals) and have been designed to act as an integrated suite to allow for data sharing between settings.[29]

interRAI assessment data are ideal to help understand the unique needs of adults with DSI across multiple countries. Individuals receiving home care or residing in a LTCF often have complex needs that are difficult to truly capture using only administrative data.[30] Two of the interRAI assessments—the interRAI LTCF and interRAI HC (home care)–have been explicitly developed to capture the needs of individuals in LTC settings, with items worded similarly or identically across the instruments. These assessments have been implemented in multiple countries, allowing for international comparisons. Looking cross-nationally at the rate of DSI, and how the needs of these individuals compare to others without DSI, we can begin to understand the implications of this unique impairment for the aging of the population and how best to target specialized services.

## Material and Methods

This paper used secondary data from four countries within the interRAI network (Canada, US, Finland, Belgium). In all cases, the assessments were collected by trained assessors, typically registered nurses, who had received training on the coding of the items using standardized terminology documented in the interRAI manuals. Assessors are instructed to complete the instruments using all sources of information, including the person, their informal caregivers, other health providers (e.g., primary care physician) and clinical records (e.g., including medical tests of hearing and vision), as available and appropriate. The data were analyzed within the home country and compiled by the authors into tables. Data protection rules in several countries preclude these data from being shared at the record level outside the home country. As such, we were unable to derive measures of statistical significance across all nations when comparing those with and without DSI. The project was reviewed and approved by the Wilfrid Laurier University Research Ethics Board.

### Measures

Several studies have established the reliability and validity of these instruments. For example, Landi et al. observed excellent agreement (Pearson’s correlation coefficient ranging from 0.74 to 0.81) between several health index scales generated within the home care instrument to established measures of functional and cognitive impairment.[31] The inter-rater reliability of five interRAI assessments, including those for home care and LTC facilities, was assessed using data from 12 countries. Across all the instruments, approximately 60% of items had a kappa value of at least 0.7, indicating excellent reliability and this was also true for both the HC and LTCF instruments.[29]

The interRAI assessments are designed primarily to support care planning and service provision and are typically captured electronically using laptop computers or tablets. The software used to capture the assessment information has embedded decision rules that minimize the possibility of entry errors (e.g., entering a value of seven for an item with response options from zero to five). Missing data are almost non-existent since the care coordinator cannot “close” and finish the assessment until all of the fields have been completed. The two interRAI assessment instruments (HC and LTCF) used here each contain approximately 300 items capturing multiple domain areas, with considerable overlap. Each assessment includes items measuring the person’s level of independence on both ADLs and IADLs, the presence of impairments in vision or hearing, the person’s capacity in both receptive and expressive communication, several items capturing indicators of negative mood, a list of chronic health conditions and several items capturing both frequency and intensity of pain. Virtually all items in these assessments are scored with a limited number of response options and typically refer to the person’s functioning in the previous three days.

Once the assessment is completed, a set of scales can be automatically generated that summarize the level of functioning for each individual across a range of issues. For example, the presence of DSI was defined using the Deafblind Severity Index (DbSI).[32] It includes two items, one each on functional hearing and vision. The hearing item asks the assessor to rate the person’s hearing, with an appliance in place (e.g., hearing aid) if that is typically used, during the previous three days. It is scored on a 4-point scale where 0 = hears adequately, 1 = minimal difficulty, 2 = hears in special situations only and 3 = highly impaired. The vision item captures the person’s ability to see close objects in adequate light while using their typical assistive device (e.g., reading glasses, magnifying glass), if required. It is scored on a five-point scale where 0 = adequate, 1 = impaired, 2 = moderately impaired, 3 = highly impaired and 4 = severely impaired. The DbSI combines these two items to create a scale that ranges from 0 (no impairment in either sense) to 5 (severe impairment in both). Previous research provides preliminary evidence of concurrent validity, as an increasing score on the DbSI has been shown to indicate both greater difficulty in performing IADLs and greater difficulty interacting with others.[32] A cut-point of 3+ on the DbSI was used to differentiate those with DSI from all others (the comparison group).

A number of other scales were also used. The Depression Rating Scale (DRS),[33] screens for signs/symptoms of depression. It is derived by creating a summary score across seven items and ranges from zero to 14. Higher scores indicate a higher degree of symptoms. The DRS correlates well with other “gold standard” clinical assessments, such as the Hamilton and Cornell depression scales. A DRS score of three or higher, as used in the current analysis, is a valid measure of clinically meaningful symptoms of depression.[33, 34] The DRS has good convergent/divergent validity and acceptable reliability in older, palliative home care clients.[35] The Cognitive Performance Scale (CPS),[36] captures issues with memory, independence in eating and decision-making. Validity has been established against the Mini-Mental State Exam (MMSE).[37, 38] The Activities of Daily Living Self-Performance Hierarchy scale (ADL-S)[39] combines multiple ADL items and gives a higher weighting to late loss ADLs (e.g., eating) to summarize the progressive loss in independence. Its validity was established with the Barthel ADL Index.[31] For home care, there is also an IADL Capacity Scale that summarizes difficulty in doing housework, preparing meals and using the telephone. It can range from 0 (no difficulty) to 3 (difficulty on all three IADLs) and is highly correlated with the Lawton IADL Scale.[31] The Pain Scale[40] uses two items relating to frequency and the intensity of pain to create a 4-point scale (0 = no pain to 3 = severe daily pain) and correlates well with the Visual Analog Scale (VAS). The Changes in Health, End-Stage Disease and Signs and Symptoms (CHESS) scale measures health instability and is strongly associated with mortality.[41]

### Data Collection

Within each participating country, there was some variability in terms of the methods used for data collection and the types of individuals assessed with the interRAI instruments. These have been summarized in Table 1.

**Table 1:** Overview of data from four countries.

	Canada	US	Belgium	Finland
Data collection	• Assessments completed by trained clinicians in home care/LTC facilities	• Assessments completed by trained clinicians in home care/LTC facilities	• Assessments completed by trained clinicians in home care/LTC facilities	• Assessments completed by trained clinicians in home care/LTC facilities
	• Typically completed by registered nurses	• Typically completed by registered nurses	• Typically completed by registered nurses	• Typically completed by registered nurses
Participants	• In home care, all clients expected to receive service for at least 60 days and mandated for all residents in LTC facilities	• Home care clients supported through the state-funded (Medicaid) home care system for aging individuals and persons with disabilities	• Home care clients who receive services that participate in home care projects for keeping older people at home longer	• All clients who receive home care services regularly on long-term basis. Temporary clients excluded
		• All residents in a LTC facility	• Home care clients of organizations that participated in implementation projects	
Geographic region represented	• Entire province of Ontario	• Entire state of Michigan	• Organisations spread over Belgium, but not the entire country	• Representative data from 8 out of 10 largest cities. Covers 20% of the home care clients in the country
Number of assessments completed annually	• Approximately 250,000 in home care	• Approximately 27,000 in home care	• Differs between years	• Approximately 35,000 assessments completed annually
	• Approximately 339,000 in LTC facilities	• Approximately 90,000 in LTC facilities	• More every year, as implementation continues across the country	
Missing data	• Negligible since assessments completed electronically	• Negligible since assessments completed electronically	• Negligible since assessments completed electronically	• Negligible since assessments completed electronically

### Data from Canada

In Canada, the interRAI assessments used in LTCFs and home care have been mandated by the government of Ontario. Although other regions are using these assessments, we focused on Ontario since it has the longest history of mandatory use of these instruments, it has the largest number of completed assessments and the data are being used to direct policy in terms of funding and public reporting on quality indicators and are being used for regulatory purposes (e.g., inspections in LTCFs). [30] Although other provinces have implemented the LTCF and home care assessments, their implementation began later so that large samples were not available for both sectors in the study period.

### Data from the US

Data were analyzed from the State of Michigan since this state has a long history of implementation of these instruments. The state-funded (Medicaid) home care system for aging individuals and persons with disabilities is designed to be an alternative to nursing home care. This program has been using interRAI assessments since 2002. At the same time, the federal government has mandated an interRAI assessment that is used nationwide in nursing homes. These two data sets were used to represent the United States in these analyses.

### Data from Belgium

Data from Belgium originate from the BelRAI database. Belgium is preparing for full implementation of the HC and LTCF instruments, and a number of implementation and test projects financed by the federal government have generated the data used.

### Data from Finland

The data from Finland originates from the RAI database, held by the National Institute for Health and Welfare (THL), a national organization with responsibility for keeping national social, and health registers and that acts under the Ministry of Welfare and Health. In Finland, the governance system in social and health care is decentralized into 301 relatively independent local municipalities and 209 distinct collaborative areas, on mainland Finland. To date, one in three of these has mandated interRAI systems, including all ten of the largest cities. Individual assessments are performed at least every six months by nurses who have received official training how to perform an interRAI assessment. The electronic copies of the assessments are collected twice annually to THL. The database comprises data from both public and private health care providers.

### Analysis

The characteristics of individuals receiving home care services, or residing in an LTCF, were summarized using individual items from within an interRAI assessment or from the health scales embedded within the instruments. Frequencies were generated for those with DSI and all others, by nation. Since some of the samples represent a near census of the population, and given the large sample sizes within each nation, almost any comparison would result in a statistically significant difference. As such, we focused on the proportions within each country and did not generate statistical test scores nor p-values. Given the large number of variables available for analysis, the reported results focus on those that represent important differences, typically, differences of at least 10%. For the majority of the comparisons being made, there is no established cut-off for what would be considered an important difference. Our choice of 10% was chosen to provide a useful number of comparisons that would match this criterion. For example, if we had chosen a lower threshold, say 5%, virtually all of the comparisons would show this level of difference. On the other hand, raising the cut-point to 20% would eliminate all but a very small number of variables (less than 10). The 10% cut-point was therefore a compromise between these options.

To address mental health issues within the DSI population,[7, 14, 16, 21] two separate multivariate logistic regression models (one for each unique setting) were used to understand the factors influencing the risk of depression. All variables and covariates were considered in each of the two models, with their respective cut-points. The covariates considered included: age (as a continuous measure), sex, a measure of social interaction (time spent alone in home care and time involved in activities with the facility in the long-term care sample), cognitive impairment (categorized as no impairment/mild to moderate/ severe), ADL impairment (categorized as none/mild to moderate/severe), and a count of co-morbid chronic health conditions (categorized as 0–2 conditions, 3–4 conditions and 5+ chronic health conditions), along with a dichotomous (yes/no) indicator of DSI (based on the DbSI, as discussed earlier). In the home care model only, an additional variable was added that assesses the degree of IADL impairment (categorized as none/minimal/moderate/severe); these measures were not assessed in the LTCF instrument. Within each nation, odds ratios, and corresponding 95% confidence intervals, were used to gauge statistical significance.

## Results

### Home Care

The prevalence of DSI among home care clients ranged from 13.4% in Finland to 24.6% in Belgium. Across all four national samples, there was little difference between DSI and all others on sex and marital status. However, those with DSI were much more likely to be aged 85+, or to have any type of psychiatric diagnosis, coronary artery disease, cataract, glaucoma, or diabetes. In terms of functional ability and health status, individuals with DSI were more likely than all others to have impairments on nearly every factor that was measured. For example, they were more likely to experience moderate to severe cognitive impairment (between 3.8% and 9.0% higher rate), highly compromised independence with ADLs (5.2% to 11.4% higher) or instrumental ADLs (8.8% to 21.4% higher), unsteady gait (7.8% to 17.9% higher) and falls (1.6% to 7.8% higher). They were also more likely to experience other negative outcomes such as poor/fair self-rated health, primary caregiver feeling overwhelmed or distressed, and bladder/bowel incontinence. As expected, they were also much more likely to experience difficulties in both understanding others (10.3% to 18.2% higher) and in expressing themselves (6.8% to 13.6% higher). Despite this level of impairment, those with DSI were not more likely to experience social isolation (Table 2).

**Table 2:** Demographic characteristics, health and functional status of home care clients comparing those with and without DSI across multiple countries.

	Canada		US		Belgium		Finland	
	Comparison N = 444,559	DSI N = 75,834 (14.5%)	Comparison N = 7,429	DSI N = 1,361 (15.5%)	Comparison N = 4,049	DSI N = 1,323 (24.6%)	Comparison N = 13,689	DSI N = 2,116 (13.4%)
	%							
Age group								
0–44	4.5	0.8	6.4	1.8	0.6	0.5	0.7	0.1
45–54	5.9	1.1	11.3	3.4	0.7	0.1	2.0	0.2
55–64	10.2	2.6	19.4	9.0	5.2	2.6	6.2	1.6
65–74	16.8	7.4	21.7	17.4	17.1	11.3	15.3	4.8
75–84	35.3	30.9	25.2	28.8	49.2	44.1	40.7	29.5
85+	27.3	57.3	16.0	39.5	27.2	41.4	35.1	63.9
Female	63.0	63.8	69.0	71.0	69.9	65.8	70.7	70.8
Married	41.3	31.9	21.3	20.3	35.4	39.7	18.2	18.1
Diagnoses								
Any psychiatric*	25.5	29.7	5.9	57.5	20.0	23.3	19.4	13.3
Coronary artery disease	22.8	29.1	31.4	40.0	8.2	13.3	23.9	31.5
Alzheimer’s disease	7.4	8.7	7.0	9.9	12.3	15.5	23.1	24.2
Cataract	13.3	25.5	1.2	1.6	1.9	1.2	6.1	12.6
Diabetes	24.1	25.5	40.0	40.8	17.2	17.8	28.0	26.0
Glaucoma	5.7	13.6	2.0	3.7	0.7	1.3	7.4	17.6
Cognitive Performance Scale (CPS)								
0	50.3	25.7	33.5	14.2	48.5	22.7	33.7	20.1
1–2	37.6	52.4	40.8	48.4	25.5	34.8	53.7	59.4
3–4	7.8	13.0	17.9	22.5	19.2	26.7	7.9	12.0
5–6	4.4	8.9	7.7	14.9	6.8	15.8	4.7	8.5
Activities of Daily Living Self-Performance Hierarchy Scale (ADL-S)								
0	64.9	46.6	34.8	25.1	26.5	20.2	73.0	61.5
1–2	22.1	30.9	30.9	34.5	27.1	22.0	16.9	23.2
3+	13.0	22.5	40.4	40.4	46.4	57.8	10.1	15.3
Walks independently	33.8	15.6	40.2	28.0	55.5	44.7	29.5	15.3
Bathes independently	29.8	13.6	11.1	6.2	13.7	10.5	41.3	25.7
Pain Scale								
0	36.1	34.0	29.9	28.0	49.9	45.4	40.7	32.0
1–2	50.9	54.2	55.0	58.0	40.8	45.0	52.0	58.7
3+	13.0	11.8	15.0	14.0	9.2	9.6	7.3	9.3
Changes in Health, End-Stage Disease and Signs and Symptoms (CHESS)								
0	31.3	24.4	30.2	20.8	39.8	37.9	49.2	34.4
1–2	55.0	56.9	61.5	64.8	51.0	47.6	43.6	52.3
3+	13.7	18.7	8.4	14.4	9.3	14.5	7.2	13.3
Depression Rating Scale (DRS)								
0	61.9	54.5	57.9	48.9	51.9	36.8	66.6	57.1
1–2	22.9	26.1	25.1	29.5	23.0	27.1	20.6	24.8
3+	15.2	19.3	17.0	21.6	25.1	36.1	13.7	18.1
Instrumental Activities of Daily Living Capacity Scale								
0	6.9	1.2	0.5	0.2	1.8	0.5	13.1	4.2
1–2	21.9	10.0	4.2	2.1	13.2	8.0	34.3	23.2
3–4	21.9	18.1	19.0	12.4	36.1	28.9	20.7	24.4
5–6	49.3	70.7	76.4	85.2	48.9	62.6	31.9	48.2
Any falls in last 90 days	30.8	38.2	24.5	26.1	36.8	37.9	22.9	30.7
Unsteady gait	56.0	73.9	66.3	74.1	56.9	70.2	64.4	81.7
Fair/poor self-rated health	19.6	22.7	59.3	60.8	62.0	67.1	31.0	41.7
Occasional or worse incontinence								
Bladder	25.3	40.5	47.1	58.4	44.6	64.4	29.3	40.3
Bowel	9.3	16.7	18.2	27.3	15.0	27.7	7.4	11.1
Hallucinations/ delusions	3.3	5.8	3.8	7.6	4.7	9.3	7.4	8.2
Social isolation	15.7	13.6	27.0	21.7	21.7	19.9	5.1	6.2
Caregivers distressed/overwhelmed	17.3	25.1	13.7	20.1	17.0	26.2	8.5	12.7
Communication impairment								
Expression	9.5	19.1	12.1	22.4	9.7	23.3	9.8	16.6
Comprehension	9.9	22.8	11.5	27.3	11.7	29.9	10.3	20.6

*Presence of any type of psychiatric diagnosis (e.g., depression, anxiety disorder, schizophrenia, paranoia)

When looking across countries, Belgium stands out within the home care sample with the highest rates (i.e., a greater degree of difficulty/impairment) on multiple factors including the presence of symptoms of depression, cognitive impairment, difficulties with ADLs, incontinence, difficulties with communication and they had the highest rates of caregivers feeling distressed. While each of the other countries had at least one variable in which they had the highest rates, none of the others showed this type of consistent pattern.

### Long-term Care Facilities

Among residents of LTCFs, the prevalence of DSI ranged from 9.7% in the US to 33.9% in Belgium. Similar to the home care population, there were only slight differences in terms of sex and marital status and residents with DSI were more likely to be 85+ (19.9% to 32.5% higher). Those with DSI were more likely to have a diagnosis of Alzheimer’s disease (1.6% to 10.1% higher) and were only slightly more likely to have a diagnosis of glaucoma (1.3% to 8.3% higher) or cataract (0.6% to 6.2% higher) (Table 3). Across the vast majority of other chronic health conditions, there were only minor differences between groups (data not shown).

**Table 3:** Demographic characteristics, health and functional status of LTCF residents comparing those with and without DSI across multiple countries.

	Canada		US		Belgium		Finland	
	Comparison N = 138,650	DSI N = 48,210(25.8%)	Comparison N = 60,971	DSI N = 6,577(9.7%)	Comparison N = 500	DSI N = 256(33.9%)	Comparison N = 4,765	DSI N = 1,358(22.2%)
	%							
Age group								
0–44	0.7	0.2	1.4	0.3	0.2	0.0	0.2	0.0
45–54	1.8	0.4	3.8	0.9	1.6	0.0	0.5	1.0
55–64	4.5	1.1	9.4	2.3	3.0	1.9	3.2	0.3
65–74	10.2	3.9	17.3	6.0	11.0	8.9	13.6	2.9
75–84	32.4	22.2	33.5	23.4	43.5	29.2	39.2	24.5
85+	50.5	72.1	34.6	67.1	40.1	60.0	43.3	72.3
Female	67.2	67.9	66.8	68.6	74.9	78.9	70.9	76.2
Married	14.7	14.1	28.2	22.5	20.0	20.1	22.6	17.2
Diagnoses								
Alzheimer’s disease	15.7	18.4	9.9	19.9	16.1	26.2	42.5	44.0
Cataract	10.4	15.8	3.5	9.7	3.7	4.3	2.9	4.9
Glaucoma	6.3	10.4	5.7	14.0	0.2	1.5	5.3	9.1
Cognitive Performance Scale (CPS)								
0	16.6	4.8	36.9	11.1	23.9	5.9	4.9	1.8
Impaired	83.4	95.2	63.1	88.9	76.1	94.1	95.1	98.2
Activities of Daily Living Self-performance Hierarchy Scale (ADL-S)								
0	7.1	2.7	2.4	1.2	10.1	3.1	2.4	1.1
1–2	18.3	10.9	19.9	11.6	15.0	8.6	13.7	9.2
3+	74.6	86.4	77.7	87.2	74.9	88.3	83.9	89.7
Walks independently	21.9	11.8	4.9	3.8	37.4	20.7	14.3	8.4
Bathes independently	0.8	0.2	0.8	0.2	2.6	1.6	0.3	0.0
Pain Scale								
0	52.8	50.0	38.7	52.0	60.6	55.7	52.9	50.0
1–2	43.3	46.0	57.2	46.2	34.5	41.6	43.9	46.9
3+	3.9	4.0	4.1	1.8	4.9	2.7	3.2	3.1
Changes in Health, End-Stage Disease and Signs and Symptoms								
0	41.7	31.7	19.5	24.0	54.1	51.6	39.0	30.6
1–2	46.4	50.3	62.4	59.3	39.0	39.0	46.0	46.9
3+	11.9	18.0	18.0	16.7	6.9	9.3	15.0	22.5
Depression Rating Scale (DRS)								
0	37.4	30.2	62.1	55.8	32.7	27.9	37.8	34.2
1–2	33.0	36.1	29.3	33.3	30.6	29.5	35.0	37.4
3+	29.6	33.7	8.6	10.8	36.7	42.6	27.2	28.4
Any falls in last 90 days	15.3	15.7	42.7	46.2	28.1	21.6	6.8	6.4
Unsteady gait	33.7	33.2	40.9	34.9	59.9	67.8	25.7	25.9
Occasional or worse incontinence								
Bladder	67.3	79.8	43.4	67.2	69.3	89.5	84.2	88.7
Bowel	40.9	54.9	31.5	52.4	43.3	70.3	71.4	47.2
Any aggressive behaviour	40.8	49.4	7.6	12.5	34.0	44.3	47.1	47.2
Communication impairment								
Expression	23.0	40.1	11.0	31.8	24.0	48.8	34.7	49.3
Comprehension	23.6	44.6	12.0	37.4	23.0	56.7	36.0	56.9
Physical restraint used	17.1	26.2	1.3	3.1	12.7	26.1	28.1	30.0

As seen in home care, LTCF residents with DSI were also more likely to experience moderate to severe cognitive impairment (13.6% to 18.4% higher), severe impairment in ADLs (5.8% to 13.4% higher) and bladder incontinence (4.5% to 23.8% higher) (Table 3). These residents had much higher rates of difficulty with both expressive (14.6% to 24.8% higher) and receptive (20.9% to 33.7%) communication.

In contrast to the home care sample, Finland appeared to have the most impaired DSI population with the highest rates of dysfunction, across the largest number of factors. For example, its residents were more likely than other nations to have Alzheimer’s disease and the highest rates of cognitive impairment, difficulties with ADLs, health instability, difficulties with communication and use of restraints. As was seen in the home care sample, Belgium again had the highest rates among LTCF residents of both symptoms of depression and issues with incontinence.

### Predictors of Depression

In both HC and LTCF, the presence of symptoms of depression was slightly higher among those with DSI, ranging from 4.1% to 11.0% higher in HC, and 1.2% to 5.9% higher in LTCFs (Tables 4 and 5). In a multivariate model, the presence of DSI had a somewhat inconsistent relationship with depression, with adjusted odds ratios (ORs) ranging from 1.08 to 1.44 in home care and from 0.84 to 1.18 in LTCFs. In both care settings, clients with severe cognitive impairment had a significant increase in the risk of depression (adjusted OR ranged from 1.71 to 4.67), even after adjusting for the other covariates in the model. The same was also true for the presence of five or more chronic health conditions (adjusted OR ranged from 1.12 to 2.96). Being female also increased the risk (adjusted OR: 1.30 to 1.88) and was significant across most settings, with the exception of LTCFs in Belgium. A reduced level of social engagement also increased the risk of depression and was significant in all but the US home care population (adjusted OR: 1.08 to 2.30). Age was not significantly related to depression and several other factors showed mixed results (e.g., ADL and IADL impairment).

**Table 4:** Results of the multivariate regression model to predict the presence of symptoms of depression in home care.

Covariate	Canada	US	Belgium	Finland
	OR (95% CI)			
Age	0.98 (0.98, 0.98)	0.98 (0.97, 0.98)	0.97 (0.97, 0.98)	0.98 (0.98,0.98)
Sex (Ref: Male)				
Female	1.41 (1.39, 1.44)	1.85 (1.71, 2.00)	1.39 (1.23, 1.57)	1.93 (1.72,2.16)
Length of time alone during the day (Ref: Never/hardly ever)				
About one hour	0.99 (0.97, 1.02)	0.90 (0.81, 1.01)	0.53 (0.45, 0.63)	1.11 (0.88,1.39)
Long periods of time	1.13 (1.11 (1.15)	1.01 (0.92, 1.10)	0.87 (0.75, 1.0)	1.41 (1.22, 1.64)
All of the time	1.46 (1.42, 1.49)	1.07 (0.97, 1.18)	1.57 (1.34, 1.85)	1.56 (1.33,1.81)
Cognitive Performance Scale (CPS) (Ref: 0 = Intact)				
1,2	1.89 (1.86, 1.93)	2.62 (2.39, 2.86)	2.65 (2.30, 3.04)	1.83 (1.62,1.07)
3,4	2.39 (2.32, 2.46)	3.72 (3.35, 4.14)	4.24 (3.63, 4.95)	2.48 (2.08,2.99)
5,6	1.82 (1.76, 1.89)	3.01 (2.61, 3.47)	4.59 (3.68, 5.72)	2.85 (2.31,3.52)
ADL Self-Performance Hierarchy Scale (Ref: 0 = Independent)				
1,2	1.23 (1.20, 1.25)	0.87 (0.80, 0.95)	0.61 (0.52, 0.72)	1.25 (1.10,1.42)
3+	1.24 (1.21, 1.27)	0.89 (0.81, 0.97)	0.78 (0.67, 0.91)	1.47 (1.25,1.72)
IADL Capacity Scale (Ref: 0 = Independent)				
1,2	1.41 (1.35, 1.47)	1.56 (0.85, 2.85)	0.93 (0.61, 1.42)	1.90 (1.53,2.35)
3,4	1.76 (1.68, 1.84)	1.45 (0.81, 2.60)	0.90 (0.60, 1.36)	2.42 (1.94,3.02)
5,6	2.27 (2.17, 2.37)	1.55 (0.87, 2.78)	0.85 (0.56, 1.29)	2.75 (2.20,3.45)
Deafblind Severity Index (DbSI) (Ref: 0,1,2 = No DSI)				
3+	1.08 (1.04, 1.12)	1.45 (1.33, 1.60)	1.21 (1.08, 1.37)	1.27 (1.12,1.44)
C statistic	0.653	0.659	0.719	0.672

**Table 5:** Results of the multivariate regression model to predict the presence of symptoms of depression in LTCFs.

Covariate	Canada	US	Belgium	Finland
	OR (95% CI)			
Age	0.99 (0.99, 1.00)	0.98 (0.98, 0.99)	1.00 (0.98, 1.01)	0.99 (0.98,0.99)
Sex (Ref: Male)				
Female	1.44 (1.41, 1.47)	1.59 (1.49,1.69)	1.30 (0.90, 1.89)	1.58 (1.45,1.71)
Average time involved in activities (REF: Greater than 2/3 of the time)				
Some—from 1/3 to 2/3	1.02 (0.99, 1.05)	0.97 (0.81, 1.16)	1.00 (0.62, 1.63)	1.25 (1.12,1.40)
Little—less than 1/3	1.28 (1.24, 1.32)	1.31 (1.04, 1.64)	1.03 (0.64, 1.66)	1.50 (1.35,1.66)
None	1.37 (1.31, 1.44)	2.30 (1.21, 4.36)	1.80 (1.03, 3.17)	1.42 (1.25,1.60)
Cognitive Performance Scale (CPS) (Ref: 0 = Intact)				
1,2	1.62 (1.56, 1.68)	2.12 (1.95, 2.30)	2.60 (1.50, 4.50)	1.49 (1.23,1.82)
3,4	2.25 (2.17, 2.34)	3.45 (3.19, 3.72)	2.76 (1.61, 4.75)	2.19 (1.81,2.65)
5,6	1.48 (1.42, 1.54)	2.24 (2.00, 2.50)	1.96 (1.08, 3.55)	1.51 (1.24,1.81)
ADL Self-Performance Hierarchy Scale(Ref: 0 = Independent)				
1,2	1.34 (1.27, 1.42)	0.90 (0.74, 1.09	1.45 (0.64, 3.29)	1.32 (1.08,1.58)
3+	1.81 (1.72, 1.91)	0.95 (0.79, 1.13)	1.36 (0.65, 2.85)	1.18 (0.98, 1.42)
Count of co-morbid health conditions (Ref:0 to 2)				
3,4	1.20 (1.17, 1.23)	1.04 (0.97, 1.12)	2.23 (1.57, 3.29)	1.19 (1.11, 1.29)
5+	1.41 (1.37, 1.44)	1.12 (1.04, 1.21)	2.96 (1.21, 7.24)	1.53, (1.38, 1.70)
Deafblind Severity Index (DbSI) (Ref: 0, 1, 2 = No DSI)				
3+	0.84 (0.81, 0.87)	1.12 (1.02, 1.22)	1.01 (0.71, 1.43)	1.18 (1.08,1.29)
C statistic	0.612	0.646	0.655	0.602

## Discussion

The prevalence of DSI in this study ranged from 10% to 34% and has a frequency similar to that of several other chronic conditions more often considered in studies of this population, such as diabetes,[42] major depression,[43] Alzheimer’s disease,[44] chronic obstructive pulmonary disease[45] and heart failure.[46] The prevalence rates reported here are somewhat higher than some earlier studies. This likely reflects the variation in definitions used to identify DSI and the fact that some previous studies have relied on self-report among older adults living in the community.[10, 12] Our study samples included older adults with compromised health who were receiving formal health care services, or were residing in a LTCF, assessed by trained health care professionals. These factors also would explain the somewhat higher rates in the current study.

Our results showed that those with DSI had higher rates of cognitive impairment, which is in line with several previous studies. For example, Lin et al. [22] reported that those with DSI had a roughly two-fold increase in the odds of cognitive decline (OR = 2.19; confidence interval: 1.26–3.81), even after adjusting for other factors such as age, medical comorbidities, smoking and walking speed. A study of older Australians also found a higher rate of cognitive impairment among those with DSI compared to those without (6.2% vs. 1.1%) of the comparison group (p<0001).[47] Similar results have been reported in Iceland showing a roughly 30% higher rate of cognitive impairment among older adults with DSI.[13]

In our multivariate models, we found increased odds of depression among the home care clients, even after adjusting for other factors known to be associated with depression. Several other studies have shown similar results and in each of these, there was a significant increased risk of depression for individuals with DSI, even after adjusting for a wide range of other covariates (e.g., age, sex, functional impairment, cognitive status, degree of social engagement). [12, 16, 48] In our sample of LTCF residents, DSI had mixed results in the multivariate model and was not significant across all countries. However, the presence of severe cognitive impairment and the level of social engagement were significant in this group, after adjusting for other factors. This is consistent with previous research showing that European LTCF residents with a combination of DSI and low levels of social engagement had the highest rates of cognitive decline.[19]

We found high rates of communication difficulties and cognitive impairment among those with DSI, which has important implications for the provision of health care and social services. Effective two-way communication is vital to ensure that the person’s goals and needs are understood by service providers and this becomes more complex in LTCFs where cognition seems to decline faster in residents with DSI than in residents with only a single sensory impairment.[49] It may also be difficult for LTCF staff to distinguish between sensory impairments and the presence of cognitive difficulties leading to the possibility that older persons with DSI may be inadvertently labeled as having dementia and vice versa.

This speaks to the need for health care organizations to create policies and practices to ensure that front-line staff has at least a basic understanding of how to assess for DSI and the appropriate next steps. A basic assessment of both functional vision and hearing should be completed, similar to what is included in the interRAI tools, as part of a comprehensive geriatric assessment. The assessment system used would ideally flag persons for whom further evaluation of their vision and hearing, and a different communication strategy, may be warranted. A thorough assessment helps professionals to better understand the needs of people with DSI and an individualized care plan can then be created to ensure the highest quality of care and quality of life for the person.

The present study has several limitations. For example, although the interRAI data represent a rich source of information, these assessments did not include details on when the person acquired the vision or hearing loss and in what order. The study sample likely had a mix of individuals with new versus existing impairments, and it was not possible to explore how the length of time with DSI influenced health and measures of cognitive and functional status.

The presence of DSI was captured based on two items within the interRAI instruments—measures of functional vision and hearing without the assurance that DSI was detected via medical testing (e.g., visual acuity test, audiometric tests). The presence of DSI was indicated by the Deafblind Severity Index (DbSI). The development and scoring of the DbSI was carried out with input from a variety of professionals working with individuals with DSI. As such, it likely captures at least a minimal functional impairment in both senses. interRAI has a Deafblind Supplement,[50] which includes about 150 items, and provides a highly detailed assessment of the person’s sensory and functional abilities. Although originally developed for use with the interRAI Community Health Assessment, it could be easily adapted for use in home care and LTCFs.

The samples in this study are not necessarily representative of the national populations in these four countries. Within each country, the data represent a particular geographic region and the samples were not randomly chosen. Therefore, the results are likely applicable only to the geographic region using the interRAI assessments and caution should be exercised in attempting to extrapolate the findings beyond these particular regions.

Research on older adults with DSI lags behind that helping us understand other complex health issues experienced in this population. More studies are needed, ideally at the national level, to understand more fully the prevalence of DSI and the needs and abilities of persons with this impairment. Organisations that work with older adults need to gather information about vision and hearing impairments since the majority of those with DSI are at least 65. Through better awareness of this disability, and by creating new policies and services to support these individuals and their families, there is tremendous potential to improve their capacity to be independent and to optimize quality of life.
